# Physical Activity, Mindfulness Meditation, or Heart Rate Variability Biofeedback for Stress Reduction: A Randomized Controlled Trial

**DOI:** 10.1007/s10484-015-9293-x

**Published:** 2015-06-26

**Authors:** Judith Esi van der Zwan, Wieke de Vente, Anja C. Huizink, Susan M. Bögels, Esther I. de Bruin

**Affiliations:** Department of Developmental Psychology and EMGO Institute for Health and Care Research, VU University Amsterdam, Amsterdam, The Netherlands; Research Institute of Child Development and Education, University of Amsterdam, Amsterdam, The Netherlands; Research Priority Area Yield, University of Amsterdam, Amsterdam, The Netherlands; Department of Developmental Psychology, Faculty of Psychology and Education, VU University Amsterdam, Van Der Boechorststraat 1, 1081 BT Amsterdam, The Netherlands

**Keywords:** Physical activity, Mindfulness meditation, Heart rate variability biofeedback, Stress, Anxiety

## Abstract

In contemporary western societies stress is highly prevalent, therefore the need for stress-reducing methods is great. This randomized controlled trial compared the efficacy of self-help physical activity (PA), mindfulness meditation (MM), and heart rate variability biofeedback (HRV-BF) in reducing stress and its related symptoms. We randomly allocated 126 participants to PA, MM, or HRV-BF upon enrollment, of whom 76 agreed to participate. The interventions consisted of psycho-education and an introduction to the specific intervention techniques and 5 weeks of daily exercises at home. The PA exercises consisted of a vigorous-intensity activity of free choice. The MM exercises consisted of guided mindfulness meditation. The HRV-BF exercises consisted of slow breathing with a heart rate variability biofeedback device. Participants received daily reminders for their exercises and were contacted weekly to monitor their progress. They completed questionnaires prior to, directly after, and 6 weeks after the intervention. Results indicated an overall beneficial effect consisting of reduced stress, anxiety and depressive symptoms, and improved psychological well-being and sleep quality. No significant between-intervention effect was found, suggesting that PA, MM, and HRV-BF are equally effective in reducing stress and its related symptoms. These self-help interventions provide easily accessible help for people with stress complaints.

## Introduction

Psychological stress, particularly persistent psychological stress, can negatively affect one’s health. Stress triggers physiological responses encompassing changes in the nervous and immune systems, such as an increased level of circulating inflammatory factors (Steptoe et al. 2007). Also, endocrine and cardiovascular systems respond to stress with, for instance, elevated cortisol levels and increased heart rate and blood pressure (Schneiderman et al. 2005). If stress is persistent, these physiological changes can result in health problems such as a (chronically) elevated blood pressure and a dysregulated immune system (Schneiderman et al. 2005), memory problems (McEwen and Sapolsky 1995), and mental illnesses such as depression (Hammen 2004).

In contemporary western societies there is a high prevalence of stress. The most recent *Stress in America™* survey showed that over two-thirds of the 2020 adult respondents from the general population experienced symptoms of stress such as fatigue, irritability or anger, or changes in sleeping habits (American Psychological Association 2013). In Europe, the *European Agency for Safety and Health at Work* reported that the average prevalence of work-related stress in 2005 in the 27 member states was 22 %, ranging from 12 % in the United Kingdom to 55 % in Greece (Milczarek et al. 2009). It can be expected that in our 24/7 society with continuous contact and interaction between people, stress levels will only increase in the coming years.

Given the high prevalence of stress, there is a critical need for effective stress-reducing methods. What is needed are interventions “that can be easily utilized by large numbers of people that are readily available, inexpensive and have minimal side effects”, as Henriques et al. (2011) stated in their paper on reducing anxiety in college students. One intervention that meets these requirements is physical activity (PA). Accumulating evidence has convincingly demonstrated the efficacy of PA in reducing stress and its related symptoms both in supervised as well as in unsupervised forms (e.g., Conn 2010a, b; Jazaieri et al. 2012; McGale et al. 2011; Pinniger et al. 2012). However, PA can cause sports injuries, and some people may not be able to carry out physical exercise due to, for instance, physical restrictions. Hence, alternative methods to reduce stress are valuable.

Two recently developed interventions with similar advantages but less physical requirements are mindfulness meditation (MM) and heart rate variability biofeedback (HRV-BF). Accumulating evidence has shown the positive influence of MM (Chiesa and Serretti 2009; Krusche et al. 2012; Pinniger et al. 2012; Wolever et al. 2012), and HRV-BF (e.g., Henriques et al. 2011; Ratanasiripong et al. 2012; Zucker et al. 2009) on psychological well-being and stress and its related symptoms.

Additional advantages of PA, MM and HRV-BF are that they can be used in a self-directed way, at any time and without being restricted to a specific location (Cavanagh et al. 2013; Henriques et al. 2011; Jazaieri et al. 2012). This is important because many people who feel stressed, anxious or depressed are reluctant to see a specialist or go to therapy, for instance because of mental illness stigma (Rusch et al. 2011). Also, resources are limited and it is often not feasible to provide face-to-face interventions to the large number of people who would benefit from them, given that a large proportion of the western population experiences some level of stress (American Psychological Association 2013; Milczarek et al. 2009). The effectiveness of self-directed PA is well established (e.g., see Conn 2010a, b for reviews), but less is known about the effectiveness of self-directed MM and HRV-BF. Even though several studies suggested that PA, MM and HRV-BF reduce stress and its related symptoms (e.g., Chiesa and Serretti 2009; Conn 2010a; Henriques et al. 2011), to the best of our knowledge, the effectiveness of these three interventions has not yet been compared. Moreover, most studies that included PA, MM and, to a lesser extent, HRV-BF, examined these interventions in a face-to-face context. If these interventions also prove to be effective when carried out in a self-directed way, they may all provide easily accessible help for large groups of people.

The purpose of this study was to compare the effects of self-directed PA, MM and HRV-BF on perceived stress, anxiety, depression, sleep quality and psychological well-being in a sample of adults with stress complaints. Our goal was to examine whether one of these self-help interventions is most preferable for reducing stress. We hypothesized that all three interventions would reduce stress, anxiety and depression, and improve sleep quality and psychological well-being. We did not have specific hypotheses for which intervention would be most preferable for reducing stress because of the lack of previous research comparing these interventions. However, one could speculate that MM and HRV-BF may be more similar to each other in terms of outcomes than to PA, because both techniques use the focusing of attention and a calm breathing pattern in their exercises.

## Methods

In the present study we compared three active interventions of 5 weeks duration each. The Ethics Committee of the Faculty of Social and Behavioural Sciences of the University of Amsterdam in the Netherlands approved of the study. All participants gave informed consent.

### Participants and Recruitment

Participants were recruited with posters and flyers distributed throughout Amsterdam, targeting adults who suffered from stress and were willing to try to reduce their stress levels. Students were also recruited during lectures at the Faculty of Social and Behavioural Sciences of the University of Amsterdam. Participants received the training for free. Also, 50-euro gift certificates were randomly allocated to 20 participants at the end of the study. Inclusion criteria were: age between 18 and 40 years and a score of 17 or higher on the Dutch version of the 10-item Perceived Stress Scale (PSS; Cohen et al. 1983). This cut-off score, which is 1 SD below the normative mean, was chosen to ensure room for improvement, and was derived from the probability scores found by Cohen and Janicki-Deverts (2012). Exclusion criteria were being pregnant and having insufficient command of the Dutch language.

### Random Allocation to Conditions

Potential participants were randomly allocated to the PA, the MM, or the HRV-BF conditions (ratio 1:1:1) immediately upon registration and before further information about the study was sent (i.e., before agreeing to participate by signing the informed consent form). They were stratified by gender and age (18–29 or 30–40) prior to randomization. Potential participants were given participant numbers upon enrollment by independent research assistants who had no access to the randomization form. Participants received information on the condition to which they were allocated after the baseline measurements.

### Experimental Procedures

Data collection took place between December 2012 and April 2013. Participants filled out a series of questionnaires online to measure demographics and various aspects of stress and stress-related symptoms, including anxiety, depression, sleep quality and psychological well-being. Participants also filled out questionnaires on mindfulness, self-compassion, attention, executive functioning, and worrying. Results pertaining to these measures are reported elsewhere (De Bruin et al. in progress). All questionnaires were completed prior to (pre-test), directly after (post-test), and 6 weeks after (follow-up) the intervention. During the intervention period, participants kept a daily dairy about their training exercises. The preferred training was included in the demographics questionnaire.

### Outcome Measures

Depression, anxiety and stress were measured with the Dutch version of the Depression Anxiety Stress Scales (DASS; De Beurs et al. 2001; Lovibond and Lovibond 1995). The DASS-21 consists of 21 statements on three seven-item subscales: (a) depression, (b) anxiety, and (c) stress. Participants rated the extent to which each statement applied to them during the previous week on four-point Likert scales. Response options ranged from 0 (did not apply to me at all) to 3 (applied to me very much, or most of the time), for which a higher score indicated higher levels of depressive symptoms, anxiety or stress. The DASS has a clinical cut-off point of five for the anxiety scale and a clinical cut-off point of 12 for the depression scale (Nieuwenhuijsen et al. 2003). No cut-off point exists for the stress scale. Internal consistency at pre-test was sufficient to very good (Cronbach’s α for Depression = 0.88; Anxiety = 0.75; and Stress = 0.81).

The Dutch version of the Pittsburgh Sleep Quality Index (PSQI; Buysse et al. 1989) was used to measure subjective perception of both sleep quality and sleep disturbances over the past month. The PSQI consists of 19 items, addressing seven components of sleep: (a) sleep quality, (b) sleep latency, (c) sleep duration, (d) habitual sleep efficiency, (e) sleep disturbances, (f) use of sleeping medication, and (g) daytime dysfunction. Each component receives a score of 0 to 3, and a score above five on the sum of component scores represents poor sleep. Based on the component scores, Cronbach’s α was 0.66 at pre-test.

Psychological well-being was assessed using the Dutch version of the Scales of Psychological Well-being (SPW; van Dierendonck 2004; Ryff and Keyes 1995). We used the shortened 39 item version by Van Dierendonck (2004). Participants indicated to what extent they agreed to each statement on a six-point Likert scale, ranging from 1 (totally disagree) to 6 (totally agree), with higher scores indicating higher levels of psychological well-being. Internal consistency (Cronbach’s α) in the present sample was 0.92 at pre-test.

### Interventions

The three interventions consisted of a 2-h introduction meeting followed by a 5-week intervention period. During the introduction meeting information on stress, stress responses and the specific intervention was provided by experts in the particular intervention, and participants practiced the intervention technique. Participants were instructed to do daily exercises at home increasing in duration over time: week 1: 10 min/day, week 2: 15 min/day, and weeks 3–5: 20 min/day.

#### Physical Activity

During the introduction meeting of the PA condition participants carried out 20 min of physical exercise (Spinning class, i.e., high intensity indoor cycling, led by a certified spinning teacher) in order to let them experience the level of activity that was required for the PA condition. Participants were free to choose a vigorous intensity activity of their liking because a set activity may not suit all participants (Asztalos et al. 2012). Furthermore, participants could vary their activity from day to day because carrying out the same activity each day could increase the risk of sports injuries. To meet the required level of exercise intensity, participants were instructed to attain the following physical signs after a few minutes of activity: deeper and faster breathing, sweating, an increased heart rate and an increased body temperature. Each participant received a brochure with additional information on stress and the positive effects of PA in reducing stress and its related symptoms, and handouts of the presentation that was given during the meeting.

#### Mindfulness Meditation

During the introduction meeting participants took part in a workshop on guided meditation including psycho-education on how mindfulness is helpful during stressful times. The raisin exercise was practiced (beginner’s mind), as well as a sitting meditation focusing on the breath, a body scan, and mindful walking. All practices were followed by an inquiry of participants’ experiences. An experienced mindfulness trainer (SB) led the workshop. Each participant received a CD of several guided meditations (e.g., awareness of breathing meditation, body scan, and mindful movements) for their daily exercises. The weekly meditation program and the meditation practices on the CD were based on the book *Mindfulness: A practical guide to finding peace in a frantic world* (Williams and Penman 2011). Each participant received a brochure with the mindfulness meditations copied from this book (chapters 5–8), instructions for the MM exercises, and additional information on stress and meditation.

#### Heart Rate Variability Biofeedback

For the HRV-BF condition participants used the Stress-Eraser (a 510[k] premarket notification-exempt, class II medical device; Helicor, New York). This non-invasive hand-held device uses an infrared finger photoplethysmograph to measure inter-beat-intervals in the pulse rate, which are used for assessing respiratory sinus arrhythmia. When practicing with the StressEraser, users try to increase their heart rate variability by breathing at approximately six breaths per minute. During the introduction meeting, participants used the ‘breathe program’ on the StressEraser to estimate their personal breathing frequency, which maximizes heart rate variability, that is, resonance frequency (Vaschillo et al. 2006). They were instructed to use their resonance frequency as an initial breathing frequency for the daily exercises. Participants received a brochure with the instructions for the breathing exercises and additional background information on stress, HRV-BF, and how to recognize and prevent hyperventilation.

### Treatment Compliance

During the 5-week intervention, participants recorded daily whether and for how long they performed their exercises. In order to maximize compliance, an implementation plan was made during the introduction meeting in which participants scheduled a time and place for each of the daily exercises in the upcoming week. In the subsequent weeks participants filled out an implementation plan online. They also received daily reminders for their exercises via WhatsApp, text message or email with a motivating one-liner (e.g., ‘Unwind from a day of hard work with 20 min of physical exercise/mindful meditation/breathing exercises’). These were identical for all interventions (except for the reference to the type of intervention). Furthermore, student-assistants called each participant weekly in order to monitor their progress, assess possible problems, and to motivate participants to continue the practice when needed.

### Statistical Analyses

#### Preliminary Analyses

Differences between groups at pre-test were analyzed using a Pearson’s Chi square test (categorical data), a One-way ANOVA or a Kruskal–Wallis test (i.e., for normally distributed and not normally distributed continuous data, respectively). Differences between groups of participants, such as those with and without missing data, were analyzed using a Pearson’s Chi square test (categorical data), a Student’s *t* test or a Mann–Whitney U test (i.e., for normally distributed and not normally distributed continuous data, respectively). Normality of distribution of the data was tested using *z*-scores, with *z* < 3.29 being considered normally distributed.

#### Intervention Effects

The effect of time and differences between groups were assessed using generalized estimating equations (GEEs; Zeger and Liang 1986). This technique adjusts for dependency of repeated measurements within one subject, and is capable of dealing with missing data (Twisk and de Vente 2002). The outcome variables were included separately as dependent variables and time (pre-test, post-test, follow-up) was included as a categorical independent variable; the working correlation structure was set to exchangeable. Analyses for between-group differences were corrected for baseline and PA was used as the reference group.

Effect sizes of changes (Cohen’s *d*) were calculated by the mean difference (e.g., post-test minus pre-test) divided by the SD of these differences. Effect sizes 0.2–0.5 were considered small, 0.5–0.8 medium and >0.8 large (Cohen 1992). All analyses were conducted in SPSS version 21.0 and two-sided *p* values <0.05 were considered statistically significant.

## Results

### Preliminary Analyses

**Fig. 1:**
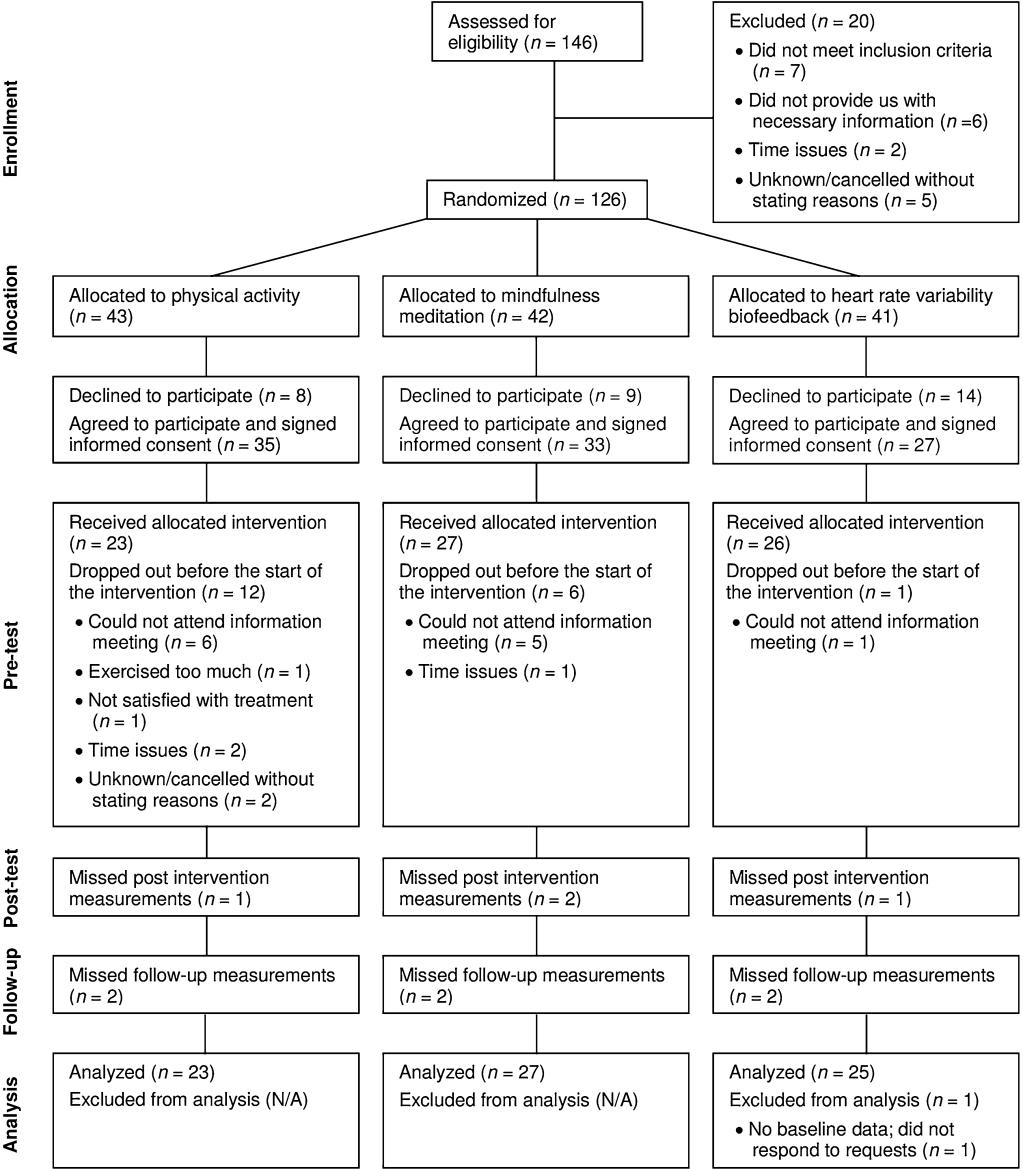
CONSORT diagram of flow of participants through the study

A summary of participant flow and loss of data is presented in Fig. 1. Potential participants who declined before giving informed consent (decliners), did not differ in stress level or age from participants who received an intervention (both *p* values >0.18). A total of 19 participants dropped out after the pre-test, but before the intervention started. The percentage of participants who were not allocated to their preferred intervention was higher for dropouts than for participants who received an intervention (*χ*^*2*^(1, *N* = 94) = 6.32, *p* = 0.012). Furthermore, 15 of the 19 dropouts reported time issues or were unable to attend the information meetings, which could also imply time issues. The participant group that received an intervention reported slightly better sleep quality at pretest than the dropout group that reported time issues (*t*(86) = −1.76, *p* = 0.082), but no other differences were found between these groups for stress and its related symptoms, age or SES at pre-test (all *p* values >0.14). Table 1 shows participant characteristics per intervention and comparative analyses of the demographic variables at pre-test. The results showed no significant differences between groups on age, gender, marital status, level of education, the amount of physical activity normally performed, and being allocated to the preferred training or not, before the start of the study.

**Table 1:** Demographic characteristics for participants randomized to PA, MM and HRV-BF

	PA (*n* = 23)	MM (*n* = 27)	HRV-BF (*n* = 25)	*F, χ* ^*2*^ or *H*	*p* value
Age, mean (*SD*)	25.28 (4.42)	26.32 (5.03)	26.99 (6.53)	*H*(2) = 0.43	0.807
Gender, *n* (%)					
Male	5 (21.74)	7 (25.93)	8 (32.00)	*χ* ^*2*^(2) = 0.66	0.720
Female	18 (78.26)	20 (74.07)	17 (68.00)		
Marital status, *n* (%)					
Relationship, living together	10 (43.48)	7 (25.93)	7 (28.00)	*χ* ^*2*^(6) = 4.53	0.605
Relationship, living apart	2 (8.70)	6 (22.22)	3 (12.00)		
Single	9 (39.13)	14 (51.85)	14 (56.00)		
Other	2 (8.70)	1 (3.70)	1 (4.00)		
Level of education, *n* (%)					
Primary school	0 (0.00)	0 (0.00)	0 (0.00)	*H*(2) = 2.39	0.304
High school	8 (34.78)	4 (14.81)	10 (40.00)		
Lower vocational school	3 (13.04)	3 (11.11)	0 (0.00)		
Higher vocational school	3 (13.04)	7 (25.93)	6 (24.00)		
University	9 (39.13)	13 (48.15)	9 (36.00)		
Min/wk PA before study, mean (*SD*)	143.93 (68.51)	199.05 (135.70)	129.63 (104.10)	*H*(2) = 3.34	0.188
Got preferred training, *n* (%)					
Yes	10 (43.48)	14 (51.85)	7 (28.00)	*χ* ^*2*^(2) = 3.11	0.211
No	13 (56.52)	13 (48.15)	18 (72.00)		

*PA* physical activity, *MM* mindfulness meditation, *HRV*-*BF* heart rate variability biofeedback, *SD* standard deviation

Eight participants missed either the post-test measurement or the follow-up measurement, and one participant in the HRV-BF group missed both the post-test and the follow-up measurements (see Fig. 1). No significant differences were found between participants with and without missing data (either during post-test or follow-up) for condition, age, gender, and the pre-test outcomes of the DASS-Stress, DASS-Anxiety, DASS-Depression or PSQI (all *p* values >0.15). Psychological well-being at pre-test, however, was lower in participants with missing data compared to participants without missing data (*t*(73) = 2.04, *p* = 0.045).

Compliance differed between interventions: the PA group reported an average exercise time that was 1.7 times longer than those reported by the MM and HRV-BF groups (i.e., 635, 364 and 375 min in total, respectively). Since self-report can be sensitive to social desirability effects, we checked whether the reported exercise time matched the actual exercise time using the reported number of points achieved by the StressEraser. Depending on the breathing frequency, participants could receive a maximum of 4.5–6 points per minute when very skilled at HRV-biofeedback. The average number of points per minute received in this study was 3.82 (*SD* = 0.89), with a maximum of 5.16. Considering that these participants were still in training, the number of points seems to be consistent with the exercise time reported. No such treatment fidelity measures were available for physical activity and mindfulness meditation, but we do not expect differences between the groups in how truthful participants were in reporting their exercise time.

The outcomes of the DASS, PSQI and SPW all showed a normal distribution at pre-test (*z* scores for skewness and kurtosis <3.29).

### Intervention Effects

Observed means and Cohen’s *d* within-group effect sizes are presented in Table 2. As can be seen, there is at least a small effect of the interventions on all outcome variables (*d*_*total*_ < −0.2 or >0.2). GEE analyses showed that stress, anxiety, depression, sleep quality and psychological well-being all changed significantly in the expected direction over time (see Table 3, left part, and Table 4, upper part).

**Table 2:** Observed means and Cohen’s *d* within-group effect sizes for stress and stress-related symptoms

Measure	Group^b^	Pre-test		Post-test		Follow-up		Pre-test–post-test		Pre-test–follow-up	
		*M*	*SD*	*M*	*SD*	*M*	*SD*	*t (df)*	*d* ^a^	*t (df)*	*d* ^a^
DASS stress	PA	16.70	8.17	11.45	8.51	11.33	9.28	3.35 (21)	−0.71	4.99 (20)	−1.09
	MM	15.41	7.92	11.52	6.91	10.08	7.43	2.44 (24)	−0.49	4.01 (24)	−0.80
	HRV-BF	13.76	6.44	11.67	6.29	10.09	6.34	1.62 (23)	−0.33	3.26 (22)	−0.68
	Total	15.25	0.87	11.55	0.85	10.46	0.92	4.32 (70)	−0.51	6.92 (68)	−0.83
DASS anxiety	PA	7.91	5.10	4.64	5.93	4.19	5.44	3.47 (21)	−0.74	3.32 (20)	−0.73
	MM	7.63	7.15	5.76	5.93	4.64	5.22	1.20 (24)	−0.24	2.43 (24)	−0.49
	HRV-BF	5.68	4.99	4.25	4.50	4.43	4.43	1.96 (23)	−0.40	1.23 (22)	−0.26
	Total	7.07	0.68	4.90	0.65	4.43	0.60	3.46 (70)	−0.41	4.05 (68)	−0.49
DASS depression	PA	10.52	7.44	5.45	7.10	7.33	7.60	3.74 (21)	−0.80	1.99 (20)	−0.43
	MM	8.07	8.03	4.80	7.00	4.64	6.47	2.06 (24)	−0.41	2.76 (24)	−0.55
	HRV-BF	6.16	6.08	6.00	5.24	5.39	5.67	0.19 (23)	−0.04	0.47 (22)	−0.10
	Total	8.19	0.85	5.41	0.76	5.71	0.79	3.58 (70)	−0.42	3.05 (68)	−0.37
PSQI	PA	5.57	2.87	5.48	3.04	5.76	2.19	0.31 (20)	−0.07	0.12 (20)	−0.03
	MM	5.89	2.87	4.64	2.12	4.96	2.77	2.34 (24)	−0.47	1.99 (23)	−0.41
	HRV-BF	5.76	2.70	5.46	2.36	4.96	2.23	0.19 (23)	−0.04	1.18 (22)	−0.25
	Total	5.75	0.32	5.17	0.79	5.21	0.29	1.79 (69)	−0.21	2.11 (67)	−0.26
SPW	PA	162.30	19.45	168.68	19.23	170.70	20.96	−2.18 (21)	0.46	−2.84 (19)	0.64
	MM	165.52	26.07	175.00	20.65	170.71	23.26	−2.64 (24)	0.53	−1.75 (23)	0.36
	HRV-BF	167.48	17.78	169.79	20.73	168.48	19.63	−1.11 (23)	0.23	−0.29 (22)	0.06
	Total	165.19	2.47	171.28	2.39	169.94	2.58	−3.48 (70)	0.41	−2.68 (66)	0.33

*M* mean, *SD* standard deviation, *df* degrees of freedom, *PA* physical activity, *MM* mindfulness meditation, *HRV*-*BF* heart rate variability biofeedback, *DASS* Depression Anxiety Stress Scale, *PSQI* Pittsburgh Sleep Quality Index, *SPW* Scales of Psychological Well-being

^a^No correction for baseline; therefore, the reported effect sizes may differ slightly from the actual effect sizes that are adjusted for regression to the mean

^b^ *n* = 19–22 PA, *n* = 22–25 MM, *n* = 23–24 HRV-BF, *N* = 64–71 Total

**Table 3:** Overall time and group effects for stress and stress-related symptoms

Measure	Within groups		Between groups^a^	
	*χ* ^*2*^ (*df* = 2)	*p* value	*χ* ^*2*^ (*df* = 2)	*p* value
DASS stress	50.64	<0.001	1.54	0.462
DASS anxiety	19.49	<0.001	1.45	0.483
DASS depression	16.42	<0.001	3.93	0.140
PSQI	4.83	0.089	3.36	0.187
SPW	14.90	0.001	2.74	0.254

*df* degrees of freedom, *DASS* Depression Anxiety Stress Scale, *PSQI* Pittsburgh Sleep Quality Index, *SPW* Scales of Psychological Well-being

^a^Corrected for baseline

**Table 4:** Estimates of stress and stress-related symptoms

Parameter	DASS stress		DASS anxiety		DASS depression		PSQI		SPW	
	*B*	*p*	*B*	*p*	*B*	*p*	*B*	*p*	*B*	*p*
*All participants (N = 75)*										
Intercept	15.25 (0.86)	<0.001	7.07 (0.68)	<0.001	8.19 (0.84)	<0.001	5.75 (0.32)	<0.001	165.19 (2.46)	<0.001
Time										
Pre-test	Ref.		Ref.		Ref.		Ref.		Ref.	
Post-test	−3.43 (0.77)	<0.001	−2.02 (0.58)	<0.001	−2.54 (0.68)	<0.001	−0.53 (0.27)	0.053	4.64 (1.25)	<0.001
Follow up	−4.86 (0.69)	<0.001	−2.64 (0.63)	0.001	−2.54 (0.78)	0.001	−0.60 (0.29)	0.040	4.74 (1.67)	0.005
*Participants with compliance levels > 70 % (N = 36)*										
Intercept	16.00 (1.28)	<0.001	8.56 (1.07)	<0.001	8.06 (1.03)	<0.001	5.83 (0.49)	<0.001	164.56 (3.42)	<0.001
Time										
Pre-test	Ref.		Ref.		Ref.		Ref.		Ref.	
Post-test	−4.42 (1.13)	<0.001	−3.23 (0.92)	<0.001	−2.52 (0.98)	0.010	−0.77 (0.36)	0.033	4.69 (1.87)	0.012
Follow up	−5.77 (0.94)	<0.001	−3.94 (1.02)	<0.001	−2.91 (1.10)	0.008	−1.23 (0.37)	0.001	7.70 (2.44)	0.002

Standard errors in parenthesis

*DASS* Depression Anxiety Stress Scale, *PSQI* Pittsburgh Sleep Quality Index, *SPW* Scales of Psychological Well-being

When considering the effect sizes of the groups separately (see Table 2) the PA intervention yielded the largest effects. MM was the only intervention that improved sleep quality. HRV-BF did not reduce depressive symptoms in contrast to the other interventions. However, note that in this group the depression score at pre-test tended to be lower compared to the PA and MM groups (Kruskall-Wallis test: *H*(2) = 5.53, *p* = 0.063). Psychological well-being also improved less in the HRV-BF group compared to the other groups. The right part of Table 3 shows that there were no statistically significant between-groups effects for any of the outcome variables (overall treatment effect corrected for baseline).

In order to (a) obtain an estimation of the potential treatment effect and (b) test whether the differences in compliance between the PA group and the MM and HRV-BF groups affected the outcome variables, we performed two sets of additional analyses. The first set of analyses were performed with a selection of participants that reported having trained at least 70 % (~7 h) of the prescribed training time, which we considered to be sufficient to expect a substantial stress-reducing effect (see Table 4, lower part). The results of these analyses showed larger regression coefficients compared to the results for the complete sample (see Table 4, upper part). This indicates that greater compliance is associated with larger effects of the interventions on stress and its related symptoms and supports a dose–response relation. As with the complete dataset, no significant differences were found between groups (all *p* values >0.07). For the second set of analyses, three subgroups were created with an equal mean training duration. This was done by removing the six participants with the longest training times from the PA group and the six participants with the shortest training times from both the MM and the HRV-BF groups. The resulting groups (PA: *M* = 449.04, *SD* = 106.29; MM: *M* = 433.67, *SD* = 49.77; HRV-BF: *M* = 445.69, *SD* = 89.49), did not differ in exercise time over the 5 weeks (*F*(2,47) = 0.158, *p* = 0.854). The results of the within- and between-group analyses for the ‘equal training duration’ groups were highly similar to the ones of the complete sample; no new significant results emerged and no significant results became non-significant (test-results available upon request).

The overall time-effects and group-effects were also analyzed with corrections for age, gender, and preferred training. Regression coefficients and *p* values of these analyses were essentially similar to the uncorrected analyses; therefore, these results are not reported here.

### Clinically Significant Change

In order to measure clinically significant change, we assessed whether there was an intervention effect on the number of participants who scored above the clinical cut-off for anxiety and depression (Nieuwenhuijsen et al. 2003), and sleep quality (indicating poor sleep; Buysse et al. 1989) at the different time points. Table 5 shows the percentage of participants that scored above the clinical cut-off for these measures. The number of participants scoring in the clinical range for anxiety was significantly reduced over time (*χ*^*2*^(2, *N* = 215) = 18.70, *p* < 0.001). No such changes were found for depression and sleep quality (*p* > 0.10). Furthermore, no significant differences were found between groups in the number of participants scoring in the clinical range for anxiety, depression or sleep quality (*p* > 0.39).

**Table 5:** Participants exceeding the clinical cut-off point for anxiety, depression or sleep quality

Measure	Group^a^	Pre-test		Post-test		Follow-up	
		%	*n*	%	*n*	%	*n*
DASS anxiety	PA	73.91	17	27.27	6	28.57	6
	MM	59.26	16	44.00	11	40.00	10
	HRV-BF	48.00	12	33.33	8	30.43	7
	Total	60.00	45	35.21	25	33.33	23
DASS depression	PA	43.48	10	18.18	4	23.81	5
	MM	22.22	6	16.00	4	12.00	3
	HRV-BF	20.00	5	25.00	6	17.39	4
	Total	28.00	21	19.72	14	17.39	12
PSQI	PA	47.83	11	47.62	10	57.14	12
	MM	40.74	11	36.00	9	37.50	9
	HRV-BF	40.00	10	41.67	10	39.13	9
	Total	42.67	32	41.43	29	44.12	30

Missing values were excluded per time-point

*PA* physical activity, *MM* mindfulness meditation, *HRV*-*BF* heart rate variability biofeedback, *DASS* Depression Anxiety Stress Scale, *PSQI* Pittsburgh Sleep Quality Index

^a^ *n* = 21–23 PA, *n* = 24–27 MM, *n* = 23–25 HRV-BF, *N* = 68–75 Total

## Discussion

The objective of this study was to compare the efficacy of self-directed PA, MM and HRV-BF on stress and its related symptoms. All interventions had substantial effects on perceived stress, anxiety, depression and psychological well-being (statistically significant), and a small effect on sleep quality (statistically non-significant). No significant between-group differences were found. Since PA is a well-established intervention, this suggests that all interventions were beneficial and that PA, MM, and HRV-BF were all equally effective in reducing stress and its related symptoms. The number of participants scoring above the clinical cut-off point for anxiety decreased (statistically significant) after the interventions.

In this study, PA, MM and HRV-BF all showed promising effects of reducing stress and its related symptoms. This adds to the body of research on the efficacy of non-pharmacologic approaches to stress treatment. The results are in agreement with self-help studies concerning PA and anxiety (e.g., Jazaieri et al. 2012), MM and stress (e.g., Krusche et al. 2012), MM and depression (e.g., Cavanagh et al. 2013), HRV-BF and anxiety (e.g., Henriques et al. 2011), and HRV-BF and stress (e.g., Ratanasiripong et al. 2012). Contrary to the study of Zucker et al. (2009), depressive symptoms did not decrease in the current study after the HRV-BF intervention. Note however, that the level of depression at pre-test in the current study was relatively low for HRV-BF, which may have caused this discrepancy in findings. An improvement in total sleep time and a trend for overall sleep improvement were found for HRV-BF in the study of Reiner (2008), but no such improvement was found in the current study. However, the analysis of Reiner (2008) only included participants with sleep problems, while in the current study good sleepers were also included. If only participants reporting poor sleep at baseline were selected in the current study, HRV-BF did improve sleep quality significantly (*χ*^*2*^(2, *N* = 10) = 20.56, *p* < 0.001). To the best of our knowledge, studies on self-help interventions are not available for PA and stress, depression and sleep quality, and for MM and sleep quality. However, the results are in line with studies using these interventions in a (partly) face-to-face context (e.g., McGale et al. 2011; Pinniger et al. 2012; Wolever et al. 2012).

Only a few studies were found that included both MM interventions and PA interventions in a face-to-face context. The study of Jazaieri et al. (2012) found that both interventions reduced anxiety and depression, and increased well-being in participants with social anxiety disorders. In line with the current study, they concluded that results for the MM and PA interventions were comparable. In the study of Pinniger et al. (2012) both meditation and tango dance lessons reduced depression in participants with self-reported stress, anxiety or depression, but only tango dance reduced stress significantly. Note that in this study, both interventions were only compared to a waitlist condition and not with each other.

In the current study, we did not find significant differences between interventions in their stress-reducing effects. However, it was only possible to detect medium to large effects with the current sample size, therefore, it is possible that smaller effects exist that were not detected in this study. In that sense, PA yielded the largest effect sizes, followed by MM and finally HRV-BF, suggesting that PA may be slightly more beneficial than MM and HRV-BF.

Compliance differed between interventions in the current study, with participants in the PA group reporting that they exercised longer on average than participants in the MM and HRV-BF groups. Greater compliance in the PA condition may have been caused by the fact that participants in the PA group were allowed to integrate their usual physical exercise activities into their daily intervention exercises. Both the MM group and the HRV-BF group had lower average compliance percentages than the 70 % compliance rate that we considered to be sufficiently high to expect a substantial stress-reducing effect. This indicates that it may be more difficult to commit to the MM and the HRV-BF interventions than to the PA intervention if one is not allowed to choose his/her intervention. The fact that participants were allowed to choose a physical activity of their liking may have made it easier to commit to the PA intervention. Furthermore, familiarity could have played a role. It may be easier to integrate something familiar, such as PA, into one’s daily schedule, than a completely new skill such as MM or HRV-BF, which these were for most participants. Overall, the results from the group with a 70 % or higher compliance rate showed larger regression coefficients compared to those from the complete sample. This indicates that greater compliance may result in higher efficacy of the interventions in reducing stress and its related symptoms. On the other hand, it could also mean that participants who will benefit more will practice more. The three interventions studied here could all play a positive role in the reduction of stress and stress-related symptoms and essentially did not differ in effectiveness when exercise duration was similar. Therefore, it may be wise to choose the intervention that is expected to be easiest to commit to (Asztalos et al. 2012).

In this study, the preponderance of female participants, the fact that most participants were relatively well-educated, and the fixed age range (18–40 years of age) limit the generalizability of the results of this study to the general population. Furthermore, long-term effects were not assessed in this study, therefore, we are not able to tell whether the effects were lasting. The results on psychological well-being may have been affected by the fact that psychological well-being at pre-test was lower in participants who missed the post-test and/or follow-up measurement compared to participants without missing data. One may suggest that participants who missed a measurement might have reported lower well-being at that time point if they had responded. Without this data, the intervention effect may have been slightly overestimated. It also suggests that it may be harder to commit to a stress-reducing intervention when psychological well-being is lower whereas these individuals might be the ones needing it most. Additionally, the 15 participants who dropped out because of time issues showed slightly poorer sleep quality at pre-test compared to participants who received the intervention. This makes sense, since a lack of time could lead to shorter sleep duration or more sleep disturbances due to for instance worrying. Finally, this study did not contain a no-treatment group, which makes it difficult to state *the extent* to which the interventions are effective. However, since PA has been proven effective with respect to reducing stress, we are confident that the effects that were found in the current study are at least partly due to the training and that the MM and HRV-BF can be considered effective.

This study shows that it is possible to obtain a substantial reduction in stress and its related symptoms using self-help interventions. There are several situations where these interventions could be useful. For example, given the long waitlists for professional help, these interventions could be offered to people who are awaiting professional help. Furthermore, about half of the people who need mental health care in Europe do not receive it (Alonso et al. 2007). This may be due to the fact that many people are reluctant to seek professional help (Rusch et al. 2011) because of financial barriers or because they think that they can work it out for themselves (Prins et al. 2011). For these people, as well as for many others, an easily accessible self-help intervention such as the interventions studied here could be beneficial.

Moreover, mental health costs are rising and self-help interventions like the ones discussed here may help reduce these costs. The World Economic Forum presented a report on current and expected global health care costs which estimated costs of mental illnesses in 2010 at nearly $2.5 trillion, and a further increase of $6 trillion is expected by 2030 (Bloom et al. 2011). Interventions like PA, MM and HRV-BF may help by reducing direct costs of mental health: they are relatively cheap, they require less face-to-face contact with a care provider, and they may even reduce the duration of untreated illnesses because of their easy accessibility. This in turn could reduce the severity of the symptoms to be treated by a professional later on. However, further research is warranted to examine the possibilities of implementing these interventions in populations with more severe problems and the possible effects of such self-help interventions on health care costs.

There seems to be a positive attitude toward alternative stress-reducing interventions. For instance, the study of Walters et al. (2008) carried out in 1383 participants attending their GP or practice nurse found that 28.7 % of participants indicated relaxation exercises or yoga as a possible help source when feeling stressed, worried, or low. This is similar to the 28.6 % indicating professional talking therapy as a possible help source. Noteworthy in the current study is that most participants preferred mindfulness meditation at pre-test (52 %, compared to 24 % for both other interventions). A possible reason for this preference for mindfulness meditation is that mindfulness is becoming more popular in Western countries, including the Netherlands. These effects may have been amplified by the relatively young and highly-educated nature of the participants in this sample. When comparing the effect over time between participants who received their preferred intervention (*n* = 31) and those who did not (*n* = 44), a difference was found for dropout (*χ*^*2*^(1, *N* = 94) = 6.32, *p* = 0.012), but not for exercise time (*t*(66) = −0.12, *p* = 0.906), nor for stress and its related symptoms (all *p* values >0.32). These results suggest that being allocated to a non-preferred intervention may affect the motivation to start the allocated intervention, but once people participate, the effort put into the training and the effects of the training are similar for people who did prefer the allocated intervention beforehand and those who did not. An explanation for this finding is that people may gain trust in an intervention once they have started the exercises.

In future studies, it may be worthwhile to further validate the treatment effectiveness of the interventions studied here with more objective measures such as fitness and HRV improvements. This is because self-report measures like the ones used in the current study are sensitive to method variance or social desirability effects. Furthermore, one could check whether being able to choose the intervention of one’s liking improves the adherence and efficacy of the interventions. In addition to that, it may be worthwhile to check whether it makes a difference if interventions are less self-directed, i.e., include more face-to-face contact with the instructor. Self-help administration with a short instruction time may not result in optimal effectiveness and it is possible that efficacy is then lower compared to more guided interventions. Such an intervention could, for instance, start with a few consecutive days of instruction or weekly appointments so that the techniques can be taught more thoroughly. One could expect that this stimulates adherence and may result in higher compliance rates. Moreover, this would provide relevant data for situations in which the interventions are given in adjunct to existing treatments because such treatments usually include regular contact with the health care provider. In addition to that, one could study whether these interventions could serve as a possible adjunct to existing treatments, or even as a possible alternative option for more common professional treatments for stress and its related symptoms.

Overall, the results of this study suggest that physical activity, mindfulness meditation, and heart rate variability biofeedback can all play a positive role in the reduction of stress and stress-related symptoms when carried out in a self-directed way. Since greater compliance is often associated with better results, the best intervention for someone may be the intervention that one finds easiest to commit to. An advantage of these self-help interventions is that they provide easily accessible help for people with stress complaints.
